# Assessment of Nuclear and Mitochondrial DNA, Expression of Mitochondria-Related Genes in Different Brain Regions in Rats after Whole-Body X-ray Irradiation

**DOI:** 10.3390/ijms21041196

**Published:** 2020-02-11

**Authors:** Serazhutdin Abdullaev, Nina Gubina, Tatiana Bulanova, Azhub Gaziev

**Affiliations:** 1 Institute of Theoretical and Experimental Biophysics, Russian Academy of Sciences, Pushchino, 142290 Moscow, Russia; nina.e.gubina@gmail.com (N.G.); gaziev.iteb@gmail.com (A.G.); 2 Joint Institute for Nuclear Research, Dubna, 141980 Moscow, Russia; bulanova_tatyan@mail.ru

**Keywords:** X-ray irradiated rats, brain regions, DNA repair, mtDNA, gene expression

## Abstract

Studies of molecular changes occurred in various brain regions after whole-body irradiation showed a significant increase in terms of the importance in gaining insight into how to slow down or prevent the development of long-term side effects such as carcinogenesis, cognitive impairment and other pathologies. We have analyzed nDNA damage and repair, changes in mitochondrial DNA (mtDNA) copy number and in the level of mtDNA heteroplasmy, and also examined changes in the expression of genes involved in the regulation of mitochondrial biogenesis and dynamics in three areas of the rat brain (hippocampus, cortex and cerebellum) after whole-body X-ray irradiation. Long amplicon quantitative polymerase chain reaction (LA-QPCR) was used to detect nDNA and mtDNA damage. The level of mtDNA heteroplasmy was estimated using Surveyor nuclease technology. The mtDNA copy numbers and expression levels of a number of genes were determined by real-time PCR. The results showed that the repair of nDNA damage in the rat brain regions occurs slowly within 24 h; in the hippocampus, this process runs much slower. The number of mtDNA copies in three regions of the rat brain increases with a simultaneous increase in mtDNA heteroplasmy. However, in the hippocampus, the copy number of mutant mtDNAs increases significantly by the time point of 24 h after radiation exposure. Our analysis shows that in the brain regions of irradiated rats, there is a decrease in the expression of genes (ND2, CytB, ATP5O) involved in ATP synthesis, although by the same time point after irradiation, an increase in transcripts of genes regulating mitochondrial biogenesis is observed. On the other hand, analysis of genes that control the dynamics of mitochondria (Mfn1, Fis1) revealed that sharp decrease in gene expression level occurred, only in the hippocampus. Consequently, the structural and functional characteristics of the hippocampus of rats exposed to whole-body radiation can be different, most significantly from those of the other brain regions.

## 1. Introduction

Ionizing radiation (IR)-induced brain damage is most often observed after radiotherapy in the management of malignant tumors of the head, neck, nasopharynx, upper jaw, pituitary gland, skull base, or metastatic brain tumors. Herewith, structures and parts of the brain located outside the irradiated area can be also damaged.

IR induces functional and morphological changes in brain tissues, vascular damage, cerebral radiation necrosis, increased oxidative stress, inhibition of neurogenesis and proliferation, changes in synoptic plasticity, decreased cognitive functions and the development of secondary brain tumors [1,2,3]. Though pronounced damage to brain tissue is usually caused by exposure to relatively high doses of IR used in radiotherapy of tumors, markedly more significant morphological and functional changes in the brain may occur from exposure to moderate- and low-level ionizing radiation [4,5].

Radiation-induced brain injury can be observed not only in patients who receive radiotherapy used to destroy tumors, but also after exposure to IR for different diagnostic and therapeutic applications. Many people receive chronic occupational radiation exposure in small doses because of working with nuclear technology. A large number of people of all age groups can be exposed to IR during radiological or nuclear incidents and accidents. Epidemiological studies of cohorts of people who survived atomic bombings or who were exposed to radiation as a result of a radiation-technical accident show that these population groups have suffered from brain dysfunctions, which persist for a long time [6,7,8]. It is known that, when total body irradiation is used, radiosensitive hematopoietic systems (primarily bone marrow), the gastrointestinal tract, and the vascular system are damaged [9].

Recently, Dietrich et al. using a granulocyte-colony stimulating factor (G-CSF) knockout receptor model of mice, reported a relationship between the bone marrow and the brain, following radiation exposure [10]. In particular, it was found that bone marrow monocytes and macrophages are necessary for the restoration of structural and functional disorders, including the regeneration of white matter, the brain and the improvement of neurocognitive functions. The authors demonstrated that bone marrow G-CSF is critical for repairing damaged brain cells [10]. Therefore, certain differences in the induction and in the restoration of brain damage are anticipated during local exposure of the skull and whole body to IR, inducing damage to the brain and blood-forming system simultaneously.

In many laboratories, active research is underway to elucidate the pathophysiological, molecular pathways and cellular effects of different doses of radiation on brain structures and their recovery. Nevertheless, the initial mechanisms of fixing the possible late effects of radiation on the brain structures are not well understood [1,3,4,5]. At present, the induction of nuclear DNA damage (nDNA) and mitochondrial dysfunctions in irradiated cells can be considered critical events leading to the cell death or development of late effects in the form of neurodegenerative diseases, oncogenesis and other human pathologies.

It is known that in postmitotic cells, different DNA repair pathways are less active than in dividing cells [11]. On the other hand, brain cell activity depends on the content of extremely high levels of ATP molecules [12]. Zhu et al. reported that a single cortical neuron utilizes approximately 4.7 billion ATPs per second in a resting human brain [12]. Meanwhile, ATP synthesis through oxidative phosphorylation (OXPHOS) in mitochondria is coupled to the generation of reactive oxygen and nitrogen species (ROS/A), which cause oxidative stress with an increase in their level in cells [13,14].

In the present work, we studied nDNA damage and repair, changes in the copy number of mitochondrial DNA (mtDNA) and the level of its heteroplasmy, as well as the expression of genes involved in the regulation of mitochondrial biogenesis and dynamics in three rat brain areas (hippocampus, cortex and cerebellum) following whole-body X-ray radiation.

## 2. Results

### 2.1. Damage and Repair of Mitochondrial DNA and Nuclear DNA

**Figure 1:**
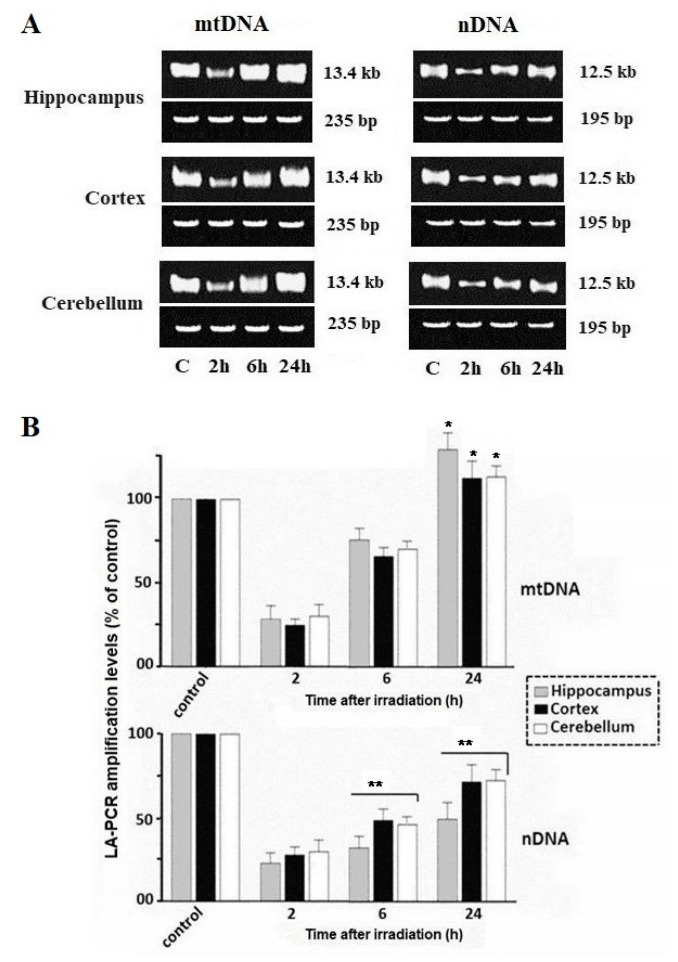
Analysis of damage and repair of mitochondrial DNA and nuclear DNA. Long fragments of mtDNA (13.4 kb) and nDNA (12.5 kb) were measured. These data were normalized by the measured levels of the short fragment of mtDNA (235 bp) and nDNA (195 bp), obtained using the same DNA sample. (**A**) Gel electrophoresis of the LA-QPCR products of nDNA and mtDNA extracted from rat brain regions at 2, 6, and 24 h after radiation exposure (here and in other figures, control: non-irradiated rats). (**B**) Quantitative analysis of the LA-QPCR amplicons of nDNA and mtDNA extracted from three rat brain regions at 2, 6, and 24 h after radiation exposure. The data are presented as mean ± SD from 4–5 independent experiments. Statistical significance was set at * *p* < 0.05, ** *p* < 0.01.

Figure 1 shows the results of the evaluation of mtDNA and nDNA damage and repair in brain regions (the hippocampus, cortex and cerebellum) of rats exposed to whole-body irradiation from X-rays. Figure 1A illustrates electrophoregrams of amplicons with mtDNA and nDNA fragments obtained after the LA-QPCR assay. Figure 1B displays the results of the quantitative analysis of the LA-QPCR products. Long fragments of mtDNA (13.4 kb) and nDNA (12.5 kb) were measured. These data were normalized by the measured levels of the short fragment of mtDNA (235 bp) and nDNA (195 bp), obtained using the same DNA sample.

It can be seen, that the level of synthesized products of the LA-QPCR nDNA and mtDNA isolated from rat brain regions 2 h after their irradiation is significantly lower compared to that of control (non-irradiated) rats. This result shows, that in irradiated rats there are nDNA and mtDNA lesions that block the DNA polymerase activity in LA-QPCR. The results of the assay also demonstrate that the number of amplified LA-QPCR products becomes greater with an addition of DNA samples, isolated from rat brain regions, into the reaction mixture 6 and 24 h after exposure. It is obvious that an increase in the amount of the synthesized product in the reactions with these DNA samples is due to a decrease in the number of lesions that reduce the DNA polymerase activity in the LA-QPCR assay. At the same time, it has been found that synthesis of amplicons with mtDNA fragments is more active when total DNA samples isolated from three rat brain regions 6 and 24 h after rat irradiation are introduced into the reaction, than that of amplicons with nDNA. It should be noted, that the synthesis of amplicons with mtDNA fragments from all three brain regions rises equally. However, as opposed to an active increase in the number of amplicons with mtDNA fragments, the number of LA-QPCR amplicons with nDNA fractions grows slower. Moreover, the findings obtained with the use of nDNA from the three brain regions are different. An increase in LA-QPCR products is statistically significant in reactions with nDNA samples from the cortex and cerebellum, whereas no such increase is seen in reactions with nDNA isolated from the hippocampus of the same rats. If an increase in the synthesis of the LA-QPCR nDNA product during the post-radiation period is due to the repair of part of nDNA lesions that inhibit DNA polymerase, it would hardly be a reason for a sharp rise in the synthesis of LA-QPCR mtDNA products from the same brain tissues. Thus, we can assume that the increase in the LA-QPCR mtDNA product is most likely induced by the post-radiation activation of mitochondrial biogenesis with mtDNA synthesis in brain tissues of irradiated rats.

### 2.2. Relative Quantification of the Total Level of mtDNA Copies

**Figure 2:**
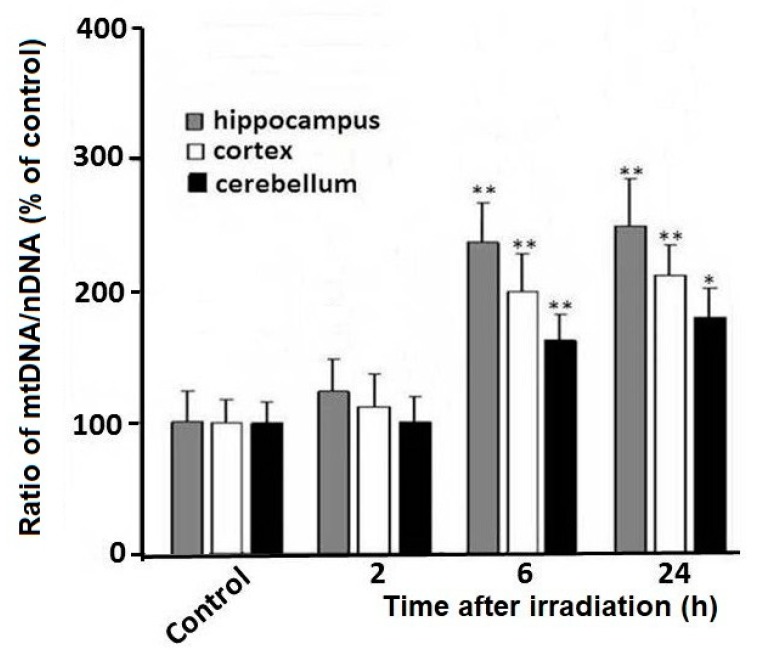
Ratio of mtDNA/nDNA in tissues of the rat brain after irradiation. The y-axis shows the percentage (%) of the change in a mtDNA to nDNA ratio relative to control. The data are presented as mean ± SD from 4–5 independent experiments. Statistical significance was set at * *p* < 0.05, ** *p* < 0.01.

Indeed, the results of real-time PCR analyses show that the mtDNA copy number increases relative to nDNA in rat brain regions (the hippocampus, cortex, and cerebellum) within 6–24 h of radiation time (Figure 2). At the same time, the mtDNA copy number doubles in the tissues of the hippocampus and cerebellar cortex. An increase in the mtDNA copy number is more pronounced in the hippocampus than in cerebellar tissue. It can be assumed that an increase in the mtDNA copy number is associated with activation of mitochondrial biogenesis. Therefore, the replicative synthesis of new mtDNA copies with the involvement of damaged matrices through DNA-Pol-γ and DNA-Pol-θ in mitochondria may lead to an increase in mtDNA heteroplasmy in the cells of the irradiated rat brain [15,16].

### 2.3. Analysis of Mitochondrial DNA Mutant Copies

**Figure 3:**
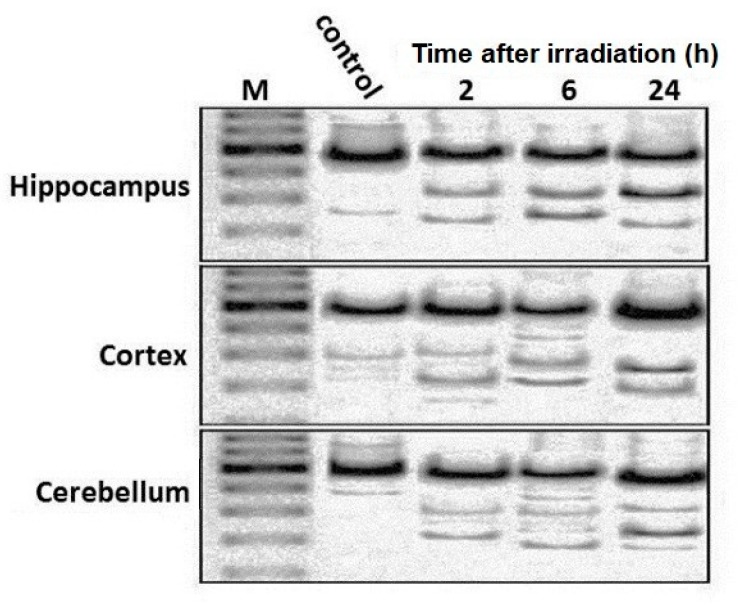
Electrophoresis of cleavage products obtained by Surveyor nucleases digestion of heteroduplexes of mtDNA PCR amplicons from the different rat brain regions at 2, 6, and 24 h after irradiation.

**Figure 4:**
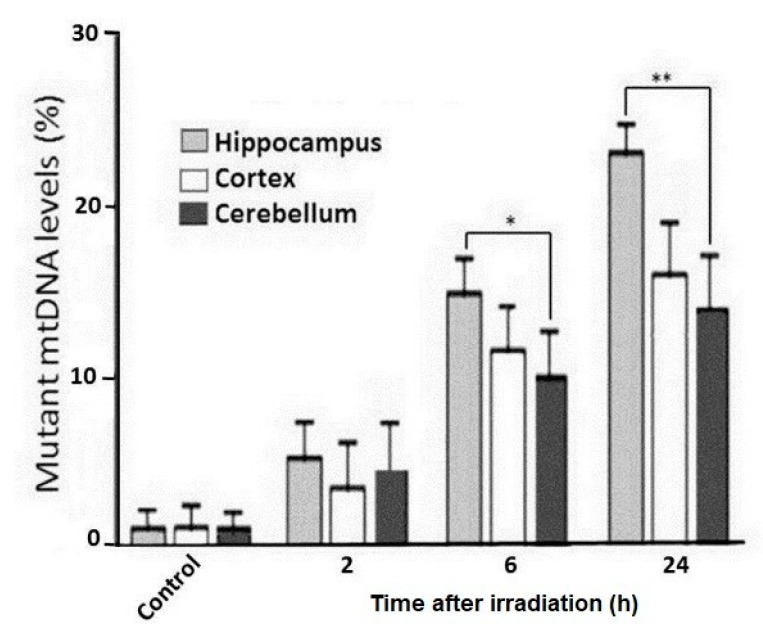
Percentage of Surveyor nuclease cleaved heteroduplexes of PCR amplicons of mtDNA (507 bp) from the different rat brain regions at 2, 6, and 24 h after irradiation. The data are presented as mean ± SD from 4–5 independent experiments. Statistical significance was set at * *p* < 0.05, ** *p* < 0.01.

Figure 3 shows the electrophoresis of cleavage products by Surveyor nuclease of heteroduplexes PCR-amplicons of mtDNA from various regions of the rat brain 2, 6, and 24 h after irradiation. It is seen that the cleavage products of heteroduplexes obtained from the PCR-amplicons of rat brain mtDNA significantly increase within 24 h after radiation exposure. Quantitative analyses showed that in all three parts of the irradiated rat brain, the copy number of mutated mtDNA increases. For instance, by 24 h after irradiation, the percentage of mutant mtDNA in the brain regions reached approximately 10–20% of the total amount of mtDNA in these tissues (Figure 4). The highest percentage of mtDNA mutant copies is observed in the hippocampus. When compared to data on the amount of mtDNA in the cortex and cerebellum, it is seen that a statistically significant increase in the copy number of mutated mtDNA is found in the hippocampus by 6 and 24 h after irradiation of the rats (Figure 4).

### 2.4. Expression Analysis of Genes Involved in Oxidative Phosphorylation, Regulation of Biogenesis, and Dynamics of Mitochondria in Three Regions of Rat Brain after Irradiation

**Figure 5:**
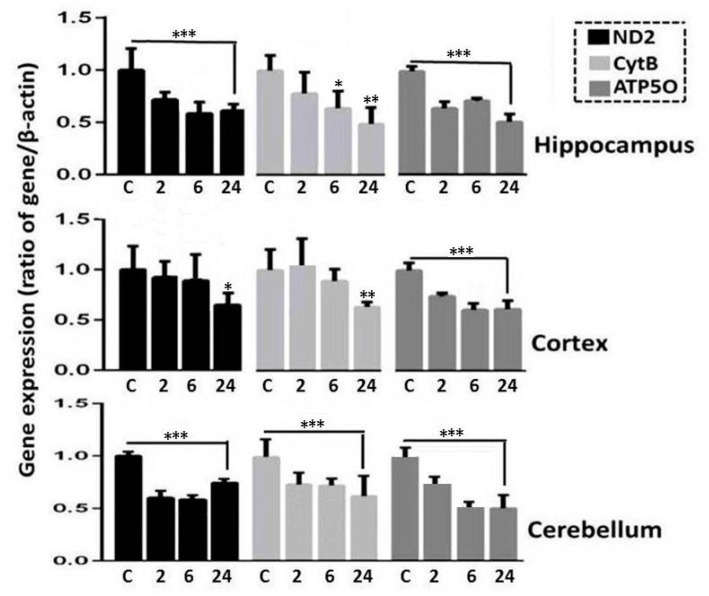
Change in the expression of oxidative phosphorylation genes (ND2, CytB, ATP5O) in the different rat brain regions at 2, 6, and 24 h after radiation exposure. The level of gene expression in unirradiated rats is expressed as 1. The data are presented as mean ± SD from 4–5 independent experiments. Statistical significance was set at * *p* < 0.05, ** *p* < 0.01, *** *p* < 0.001.

The results of expression analyses of genes involved in oxidative phosphorylation (OXPHOS), which takes place in mitochondria, in three brain regions in X-irradiated rats, are shown in Figure 5. These results include information obtained after the analysis of mtDNA-encoded gene transcripts (ND2 is a component of Complex I and CytB is a component of Complex III), as well as nuclear DNA-encoded ATP5O gene, a component of Complex V (ATP-synthase subunit).

According to the results of the analyses, a significant decrease in the expression of three genes (ND2, CytB, ATP5O) occurs in all tissues of the rat brains 24 h after their irradiation. More drastic reduction in expression of these genes in the post radiation period is seen in the hippocampus and cerebellum (Figure 5).

**Figure 6:**
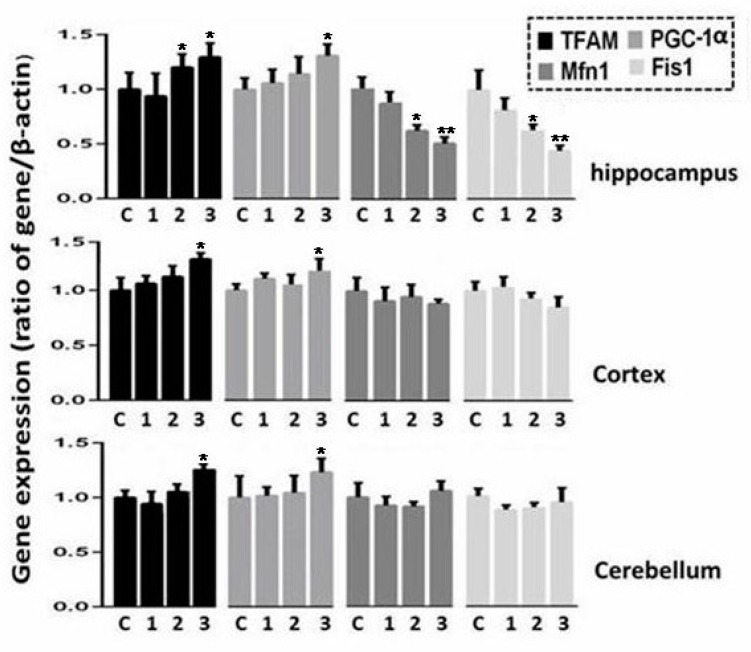
Change in expression of genes involved in biogenesis, transcription regulation (TFAM, PGC-1α) and mitochondrial dynamics (Mfn1, Fis1) in rat brain regions after radiation exposure. The level of gene expression in unirradiated rats is expressed as 1. The data are presented as mean ± SD from 4–5 independent experiments. Statistical significance was set at * *p* < 0.05, ** *p* < 0.01.

The results of expression analyses of the two nDNA genes encoding mitochondrial transcription factor A (TFAM) and peroxisome proliferator-activated receptor gamma coactivator 1-alpha (PGC-1α), which are involved in the regulation of replication, transcription of mtDNA and mitochondrial biogenesis, are presented in Figure 6. This figure also depicts the results of the analysis of the levels of gene transcripts Mfn1, Fis1 known to regulate mitochondrial fusion and division. The data obtained show that by 24 h of post radiation time, a significant increase in the transcripts of the TFAM and PGC-1α genes in three regions of the brain is observed.

As for the Mfn1 and Fis1 genes, we see a change in their expression only in the hippocampus. Our findings demonstrate that in the hippocampus of the rats, a decrease in expression of Mfn1 and Fis1 occurs at 6 and 24 h after radiation exposure.

Thus, a sharp decrease in expression of genes involved in OXPHOS in mitochondria in the three rat brain regions occurs in the rat after X-ray exposure. In addition, an increase in the activity of nuclear genes controlling mitochondrial biogenesis in the brain tissues of irradiated rats was found.

## 3. Discussion

The results of our analysis give evidence that the repair of common nDNA lesions in three rat brain regions proceeds rather slowly within 24 h after irradiation of their whole-body. It should be noted, that the repair of nDNA from the hippocampus is much slower than the repair of nDNA from the cerebellum and cortex of the same rats. These data are consistent with the results of a recent study, which showed that a delay in DNA repair in the hippocampus occurs even at significantly low doses of mouse brain irradiation [17].

Notably, if differentiated neurons attain a post-mitotic state, then glial cells (astrocytes, oligodendrocytes, microglia cells) can be proliferating and nonproliferating cells. And post-mitotic cells are characterized by a reduced level of activity of various DNA repair systems, in contrast to proliferating mammalian tissue cells [11]. In this study, we analyzed brain tissue samples containing both glial cells and neurons, and the results suggest that low nDNA repair activity may be because of the presence of neurons. Recently, it was shown that after irradiation of the rat head with X-rays, DNA double-stranded breaks (DSBs) in the cortical neurons persist for a long post-radiation time [18]. Ambrosio S et al. suggested that DNA DSBs generated in G_0_ quiescent cells are not repaired and maintain a sustained activation of the p53-pathway [19]. Previously, it was also shown that the repair of DNA DSBs and expression of genes involved in their repair by the NHEJ mechanism are insufficiently active in the rat hippocampus after cranial irradiation with a dose of up to 10 Gy [20]. The slow repair and gradual accumulation of lesions in nDNA can result in an anomaly in gene expression regulation. Unrepaired DSBs in nDNA are the cause of the neuronal death, and their misrepair leads to mutagenesis and genome instability [21]. However, there is another cause of gradual accumulation of DNA lesions in the brain cells of irradiated rats. It is the long-term (delayed) production of RONS in mitochondria that can induce lesions in nDNA and mtDNA cells following exposure to ionizing radiation [22]. Figure 1 shows that although the recovery of nDNA occurs slowly within 24 h after irradiation, mtDNA reaches higher levels in each region of the rat brain.

It is reasonable to suggest that these findings provide evidence that mtDNA lesions are repaired in the brain tissues of rats exposed to X-ray. However, it is known, that in mammalian mitochondria, only the base excision repair pathway functions efficiently [23]. Other DNA repair pathways are not effective enough in mammalian mitochondria. A series of studies has shown that after the production of mtDNA DSB, mtDNA becomes linear and degradable by exonucleases in mammalian cells. That is why no DSBs repair of mtDNA occur [24,25,26,27]. However, there are some data showing that homologous recombination is important for the maintenance of structural stability of the mitochondrial genome [28].

We believe that an increase in mtDNA content observed in our study is largely due to the activation of mitochondrial biogenesis with mtDNA replication. This assumption is consistent with the results obtained after analysis of changes in mtDNA content relative to nDNA content (Figure 2). From Figure 2, it can be seen that the copy number of mtDNA increases sharply, relative to the nDNA copy number. Our data correspond with previous studies showing that mitochondrial biogenesis and mtDNA synthesis are activated after radiation exposure in different cells (in vitro) and in rodent tissues (in vivo) [29,30]. The brain needs a lot of energy to operate [12], in this context, it can be assumed that after radiation exposure, a rapid activation of mitochondrial biogenesis in the irradiated brain is triggered for replenishment of dwindling energy levels.

It is known, that the DNA damage response (DDR) is an energy-intensive process requiring an increased consumption of ATP. So, only with the use of ATM and ATR protein kinases, more than 900 chromatin phosphorylation sites, encompassing more than 700 proteins, were identified [31]. In response to the generation of single DNA DSB, the phosphorylation of H2AX histones can encompass a chromatin region larger than one mega base pair [32]. Mitochondrial retrograde signaling to the nucleus contributes to the activation of nuclear genes that regulate mitochondrial biogenesis and restoration of neuronal functions [33].

Post-radiation activation of mitochondrial biogenesis and ATP synthesis are coupled with an increase in RONS generation, the source of which are the complexes of OXPHOS [14]. It is well known, that the increased generation of RONS persists for a long time after radiation exposure. This prolonged activation of RONS generation results in increased oxidative stress in the irradiated cells [13,22].

It is rather obvious, that if damaged copies of mtDNA are not restored or eliminated by nuclease, then their replication promotes an increase in mtDNA mutation and deletions [15,16]. This leads to mitochondrial dysfunction and increased oxidative stress.

The data obtained from our experiments show that after irradiation, the number of mtDNA mutant copies increases in all three brain regions of irradiated rats. A more significant increase in the number of mutant copies of mtDNA is observed in the hippocampus (Figure 4). It has been demonstrated in different studies, that ionizing radiation induces mutations and specific deletions in mtDNA in cells (in vitro) and rodent tissues (in vivo) [15,16]. The increased number of mtDNA mutant copies is also due to the so called ‘clonal expansion’ occurring as a result of predominant replication of mtDNA with certain types of mutations [34]. An increase in the number of mtDNA mutant copies causes disturbance in the activity of enzymes of OXPHOS complexes, leading to mitochondrial dysfunctions with increased oxidative stress, which can be involved in the development of cognitive impairments, carcinogenesis and other pathologies [35].

Alteration to the gene expression level is a major cellular response to IR. Mitochondrial biogenesis and maintenance of mtDNA depend on the expression of nuclear and mitochondrial genes. The results of our studies have shown that statistically significant changes in the expression of OXPHOS genes are observed in three brain regions of the irradiated rats. These changes are probably due to the presence of unrepaired damage, deletions and mutations in mtDNA. The expression of mtDNA genes depends on a variety of proteins, encoded by nDNA and imported into mitochondria, that regulate the maintenance of the genome, replication, transcription, and RNA maturation in these organelles [36]. It has been shown earlier in different studies that, depending on radiation dose and the time after radiation exposure, different modulations of mitochondrial genes expression can be seen in various cells and animal tissues (in vivo) [36,37]. Chien et al. reported that irradiation of hippocampal neurons at low doses leads to enhanced expression of genes encoding OXPHOS complexes I and III [38]. Apparently, a dramatic decrease in the expression of ND2, CytB and ATP5O genes in the brain regions is responsible for perturbation of OXPHOS with an increase in RONS and may induce mitochondrial dysfunction.

It should be noted that a decrease in expression of genes encoded by mtDNA occurs simultaneously with activation of a variety of genes encoded by nDNA. For instance, irradiation of the rat brain modulated expression of 1574 genes, with 855 genes among them showing more than 1.5-fold variations [39]. Prominent changes in gene expression profiles in 128 to 334 genes were seen in the rat brain after exposure to a dose of radiation up to 20 Gy. The changes were also observed in genes encoding mitochondrial OXPHOS complexes I, III, IV and V [40]. Expression analysis of genes (TFAM, PGC-1α) involved in mitochondrial biogenesis demonstrates that the activity of these genes in brain tissues increases in the post radiation period for 24 h. The results of this analysis confirm our assumption that the mtDNA copy number increases in the post radiation period, in the three brain regions, due to mitochondrial biogenesis. TFAM and PGC-1α participate in mtDNA replication and transcription and play a crucial role in mtDNA integrity [41,42]. A recent study showed that IR (at a dose of 5Gy) induces expression of PGC-1α and TFAM which promote improved metabolism and viability of different human and murine cell lines [43]. Most likely, the quantitative disorder and functional impairments in TFAM and PGC-1α may affect mitochondrial homeostasis in neurodegenerative diseases. Unfortunately, little is known about the pathways of disturbances related to the regulation of TFAM and PGC-1α in brain structures after exposure to IR.

Mitochondrial dynamics are controlled by two opposite processes: Fusion and fission. These processes maintain mitochondrial homeostasis, regulate mitochondrial shape and functions. Mitochondrial dynamics plays a key role in controlling the quality of organelles and in preserving mitochondrial functions eliminating dysfunctional mitochondria through mitophagy [44].

The results of our analyses showed, that in the cortex and cerebellum of the irradiated rats the expression levels of genes (Mfn1, Fis1) that regulate mitochondrial dynamics were unchanged for 24 h. At the same time, in the hippocampus the considerably decreased expression of these genes was observed. These changes in the components of mitochondrial dynamics in the hippocampus are probably important for preventing the elimination of dysfunctional organelles from brain cells via mitophagy [44].

In contrast to this view, Chien et al. wrote that low doses of IR may positively influence the regulation of mitochondrial quality and preservation of mitochondrial functions to provide neuronal viability [38]. Interestingly, there is an abundance of data on the important role of alterations to mitochondrial dynamics in pathogenesis of neurodegenerative diseases and on pathologies found during this process [45].

Thus, our results show that the repair of damage to nDNA in various parts of the rat brain after whole-body X-ray irradiation proceeds slowly. However, activation of mtDNA synthesis with an increased level of heteroplasmy is observed.

Obviously, an increase in the number of copies of mtDNA is the result of activation of mitochondrial biogenesis. It can be assumed that an increased level of mtDNA heteroplasmy leads to perturbation of the OXPHOS complexes, and the generation of an increased level of RONS, which induce additional damage to nDNA. A decrease in the expression of nuclear genes that regulate the dynamics of mitochondria probably contributes to a decrease in the elimination of dysfunctional organelles from brain tissue, affecting, most of all, the cells in the hippocampus. The observed changes in the brain regions of rats exposed to whole-body irradiation can lead to the development of delayed effects of radiation exposure.

## 4. Materials and Methods

### 4.1. Animals and Their Irradiation

Wistar male rats aged 2 months were obtained from the Animals Breeding Center of the Branch of Institute of Bioorganic Chemistry, Russian Academy of Sciences (Pushchino, Moscow Region). All experiments with animals followed the European Convention for the Protection of Vertebrate Animals used for Experimental and other Scientific Purposes, Directive 2010/63/EU, and the requirements approved by the administration of our institute. Rats were fed a special diet for mice and rats ad libitum, with free access to clean drinking water. The animals were irradiated at the Common Use Centre—Group of Radiation Sources at the Institute of Cell Biophysics RAS, using an X-ray apparatus RUT-250-15-1 (280 kVp, 20 mA) with 1 mm AL and Cu filters at a dose rate of 1 Gy/min. The animals were irradiated in plastic containers at dose of 5 Gy. This sublethal single dose for rats is within the dose range for assessing DNA damage and repair in experiments [18]. This dose also causes a decrease in cell proliferation in the dentate gyrus of the brain of adult rats [46] and bone marrow damage [47]. Animals were decapitated either at 2, 6, or 24 h after irradiation. Unirradiated rats were used as a control. The tissues from three regions of the brain (hippocampus, cortex, cerebellum) were separated with a scalpel on ice immediately after decapitation, frozen and stored at −80 °C until DNA and RNA isolation.

### 4.2. DNA Isolation and Purification

Frozen tissue samples from three brain regions were thawed at room temperature before isolation of total DNA (nDNA and mtDNA), and then placed on ice. Then, brain tissue samples were disrupted with the glass homogenizer and DNA isolated using the QIAGEN Genomic Tip Kit and Genomic DNA Buffer (Qiagen, Hilden, Germany). The DNA quantity in all cases was determined by reaction with the PicoGreen reagent according to the manufacturer’s protocol (Molecular Probes, USA), with the registration of fluorescence on an Infinite 200 NanoQuant device (Tecan Group Ltd., Austria). DNA samples intended for analysis of the mitochondrial genome were incubated for 20 min at 25 °C, in TE buffer with restriction endonuclease XhoI (New England Biolabs, cat.№. R0146S). XhoI endonuclease initiated a break at the site of the CTCGAG hexamer of the super-helical mtDNA of the rat, outside of the amplified fragment, leading to relaxation of mtDNA, making the selected region accessible for PCR amplification [48].

### 4.3. Analysis of Damage and Repair of Mitochondrial DNA and Nuclear DNA

To determine damage and repair of nDNA and mtDNA, we used the long amplicon quantitative polymerase chain reaction (LA-QPCR) method [49,50]. In these analyses we used (400 U; 2 U/μL) KAPA Long Range Hot Start DNA Polymerase (KAPA Biosystems), which is optimized for LA-QPCR with rat DNA [50]. The PCR primers employed in this study are given in Table 1. LA-QPCR was used to amplify a 12.5 kb region of nDNA and 13.4 kb of mtDNA. For amplification of a long fragment of mtDNA (13.4 kb), the standard thermocycler program included initial denaturation at 94 °C for 5 min, with 18 cycles of 94 °C for 30 s, and 68 °C for 12.5 min, and with a final extension at 72 °C for 10 min. To amplify a long fragment of nDNA (12.5 kb), the thermocycler profile included initial denaturation at 94 °C for 5 min, and 28 cycles of 94 °C for 30 s and 68 °C for 12 min, with a final extension at 72 °C for 10 min. Preliminary assays were carried out to ensure the linearity of PCR amplification with respect to the number of cycles and DNA concentration. Since amplification of a small region would be relatively independent of oxidative DNA damage (low probability), a small DNA fragment for nDNA (195 bp) and for mtDNA (235 bp) was also amplified for normalization of the data obtained with the large fragments, as described previously [49]. PCR analyses were performed in triplicate for each DNA sample. All of the amplified products were resolved and visualized using agarose gel electrophoresis and quantitated with an Image Quant (Molecular Dynamics) or Varsadoc (Bio-Rad) system. The data were plotted as histograms with relative amplification such as y-axis, which was calculated comparing the values of exposed samples with the control. All primers are presented in Table 1.

### 4.4. Quantitative Analysis of Mitochondrial DNA Copies Relative to the Nuclear DNA

Quantitative analysis of mtDNA was carried out by real-time PCR with TaqMan oligonucleotides on a Prism 7500 thermal cycler (Applied Biosystems, USA) [51]. The changes in the relative quantity of mtDNA in respect to nDNA were determined as a ratio between the number of copies of the mitochondrial tRNA gene and that of the GAPDH gene of nDNA in the same test tube. The 2^−ΔΔCT^ method was used for analysis. PCR tests were carried out in triplicate for each DNA sample. The PCR primers employed in this study are given in Table 1. The following PCR program was used: 5 min at 95 °C followed by 40 cycles (95 °C for 30 s, annealing and elongation at 60 °C for 1 min). The results are presented as a percentage of data compared to unirradiated rats (taken as 100%).

### 4.5. Surveyor Nuclease Assay of mtDNA Mutant Copies

To evaluate the relative level of mutant copies of mtDNA isolated from brain tissue, we used Surveyor^®^ Mutation Detection Kit (Transgenomic, Omaha, NE, USA), as described [52]. To estimate mutations in mtDNA, a region including the tRNA gene (507 bp) was chosen for amplification. The PCR primers employed in this study are given in Table 1. PCR was carried out by a programmed thermocycler (Applied Biosystems, USA). PCR was performed in a 25 µL volume containing 1.0 ng of total DNA, 75 mM Tris-HCl, pH 8.8, 20 mM (NH4)_2_SO_4_, 2.5 mM MgCl2, 200 µM of each dNTP, 250 nM of each primer, 0.01% tween-20, and 1.0 unit of total mixture of Taq- and Pfu polymerases (Thermo Scientific, Pittsburgh, PA, USA). PCR was initiated by a “hot start” after initial denaturation for 4 min at 94 °C. The amplification was carried out in 40 cycles under the following conditions: 30 s at 94 °C, 30 s at 62 °C, and 1 min at 72 °C; the final extension step of 4 min was at 72 °C. After the PCR was completed, all amplification products were diluted to an equal concentration. To obtain heteroduplex DNA, equal volumes (7 µL) of PCR products of mtDNA amplification from control and exposed rats were mixed. The mixtures were heated at 95 °C for 10 min and cooled slowly to 40 °C for 70 min at a rate of 0.3 °C/min. Then, 1/10 volume of 0.15 M MgCl_2_ solution, 1 µL of Surveyor Enhancer S and 1 µL of Surveyor Nuclease S were added to the heteroduplex mixture. The mixture was incubated at 42 °C for 60 min. The reaction was then stopped by adding 1/10 volume of stop solution. Nuclease digestion products were analyzed by electrophoresis in a 2.0% agarose gel stained with ethidium bromide. PCR tests of heteroduplexes were carried out in triplicate for each DNA sample. The fluorescence intensity of DNA bands in the gels was registered by the AlphaImager Mini System (Alpha Innotech, Santa Clara, CA, USA). The ratio of cleavage products fluorescence to the total intensity of fluorescence of DNA bands in the gel (% of Surveyor Nuclease cleaved DNA) was calculated using image software package (Wayne Rasband, NIH, USA).

### 4.6. RNA Isolation, Reverse Transcription and Real Time PCR

The total RNA from the brain tissues was extracted by RNA isolation kit ExtractRNA (Evrogen, Russia) according to the manufacturer’s instructions. RNA concentration was determined using spectrophotometer NanoVue Plus (GE Healthcare TM, Chicago, IL, USA) and adjusted to 400 ng/μL. Two μg of RNA were reverse transcribed into cDNA using MMLV reverse transcriptase (Evrogen, Moscow, Russia) according to manufacturer’s protocol in a total reaction volume of 20 μL. Real-time PCR was conducted on the machine ABI7500 (Applied Biosystems, USA) using qPCRmix-HS SYBR LowROX kit (Evrogen, Russia) following the manufacturer’s protocol. The reaction mix contained 4 μL of twenty-fold diluted cDNA and 250 nM of each primer. Primer sequences are given in Table 1. The PCR consisted of initial denaturation followed by 40 cycles of denaturation at 95 °C for 15 s, annealing at 60 °C (for ND2, CytB, TFAM, PGC-1α, Mfn1, and Fis1) or 63 °C (ATP5O, β-Actin) for 20 s, and extension at 72 °C for 40 s. Melting curves analyses were performed for all genes, and the specificity, as well as integrity of the PCR products were confirmed by the presence of a single peak. The absolute values of expression were determined by standard curve obtained as a series of 10x dilutions of pooled cDNA sample. Levels of transcription of target genes were normalized by the level of reference gene β-Actin. The normalized gene expression values were analyzed using Prism GraphPad 7.0 software. Gene expression in control rats was taken as 100%.

### 4.7. Statistical Analysis

Statistical significance was analyzed by one-way ANOVA followed by Dunnett’s multiple comparisons post-hoc test. The results were calculated and presented as a mean ± SD (*n* = 6). A value of *p* < 0.05 was considered to be statistically significant.

## Figures and Tables

**Table 1:** Primers and probes used in the current study.

Locus	Primers, Probes	5′→3′ sequence	Size, bp
		Primers for quantitative analysis of mtDNA/nDNA	
mt-tRNA	for	AATGGTTCGTTTGTTCAACGATT	
	rev	AGAAACCGACCTGGATTGCTC	
	probe	R6G-AAGTCCTACGTGATCTGAGTT-RHQ1	73
GAPDH	for	TGGCCTCCAAGGAGTAAGAAAC	
	rev	GGCTCTCTCCTTGCTCTCAGTATC	
	probe	FAM-CTGGACCACCCAGCCCAGCAA-RTQ1	80
		Primers for LA-QPCR	
mtDNA	for	AAAATCCCCGCAAACAATGACCACCC	
	rev	GGCAATTAAGAGTGGGATGGAGCCAA	13.4 kb
nDNA	for	AGACGGGTGAGACAGCTGCACCTTTTC	
	rev	CGAGAGCATCAAGTGCAGGCATTAGAG	12.5 kb
mtDNA	for	CCTCCCATTCATTATCGCCGCCCTTGC	
	rev	GTCTGGGTCTCCTAGTAGGTCTGGGAA	235
nDNA	for	GGTGTACTTGAGCAGAGCGCTATAAAT	
	rev	CACTTACCCACGGCAGCTCTCTAC	195
		Primers for mtDNA mutant copies	
mt-tRNA	for	CACACTCTCACTCGCATGAA	
	rev	TCCTTCCAATCTAGTTGAGG	507
		Primers for gene transcripts (RT-PCR)	
ND2	for	ATGGCCTTCCTCACCCTAGT	
	rev	GTTAGGGGGCGTATGGGTTC	146
CytB	for	CACGCTTCTTCGCATTCCAC	
	rev	GGGATTTTGTCTGCGTCGGA	130
ATP5O	for	GCTGAAAATGGTCGCCTAGG	
	rev	AGGAAACGCTGTGGTCAC	110
Mfn1	for	CGCCTGTCTGTTTTGGTTGA	
	rev	GCATTGACTTCACTGGTGCA	146
Fis1	for	AAAGAGGAGCAGCGGGATTA	
	rev	TGGGGCTCAGTCTGTAACAG	110
PGC-1α	for	GCACCAGAAAACAGCTCCAA	
	rev	TTGCCATCCCGTAGTTCACT	121
TFAM	for	ATCAAGACTGTGCGTGCATC	
	rev	AGAACTTCACAAACCCGCAC	115
β-Actin	for	TCTTCCAGCCTTCCTTCCTG	
	rev	CAATGCCTGGGTACATGGTG	147
